# Uncovering Correlations between Structure and Valence Tautomerism in Cobalt-o-Dioxolene Crystals

**DOI:** 10.1021/acs.jpcc.6c02263

**Published:** 2026-07-08

**Authors:** Marcelo Francis Fernandes Alecrim, Ludmila Leroy, Lucas Gustavo Gonçalves Pimenta, Lucas Olímpio Michel Machado, Majed Chergui, Simone Silva Alexandre, Carlos Basílio Pinheiro

**Affiliations:** a Physics Department, 28114 Universidade Federal de Minas Gerais, Av. Antônio Carlos, 6627, Belo Horizonte, Minas Gerais 31270-901, Brazil; b Lab. of Ultrafast Spectroscopy ISIC, FSB-BSP, École Polytechnique Fédérale de Lausanne, Lausanne CH-1015, Switzerland; c Elettra-Sincrotrone Trieste, SS 14, km 163.5, Basovizza, Trieste 34149, Italy

## Abstract

Bistability in transition-metal complexes can originate from several phenomena, including mixed valence (MV), spin-crossover (SCO), and valence tautomerism (VT). In the latter case, valence-tautomeric interconversion (VTI) generates pairs of switchable redox isomers whose physical properties can be controlled by external stimuli, such as light, temperature, and pressure, making these systems promising candidates for molecular devices, spintronics, and chemosensing. Although several strategies exist to modulate VTI in crystalline materials, tuning intermolecular interactions has emerged as a particularly effective route. In cobalt-o-dioxolene complexes of the type [Co­(dioxolene)_2_(PyL)_2_] (PyL = pyridyl-like ancillary ligands), VTI is extremely sensitive to crystal packing and the local chemical environment, and its occurrence can be promoted or suppressed by solvation. Here, we show that, in addition to the intrinsic metal–ligand distance changes across VTI, torsion of the pyridyl ancillary ligands in [Co­(dioxolene)_2_(Py)_2_] stabilizes specific electrostatic interactions and acts as a structural trigger for VTI, establishing a direct geometric-electronic coupling mechanism. This conclusion is supported by single-crystal X-ray diffraction (SCXRD) data collected on distinct solvated [Co­(dioxolene)_2_(Py)_2_] crystals, complemented by DFT calculations performed with the SIESTA package and benchmarked against experimental magnetic susceptibility data using the local density approximation (LDA) functional. DFT spin-polarization analyses reveal that the torsion angle of the PyL plane in [Co­(dioxolene)_2_(Py)_2_] correlates directly with the spin state of the system. This behavior extends to all *trans*-[Co­(dioxolene)_2_(PyL)_2_] derivatives with 3D structures deposited in the Cambridge Structural Database (CSD). Our findings establish a coherent picture of the redox and spin-state dynamics in VT complexes and provide concrete design guidelines for their use in practical solid-state applications.

## Graphical abstract

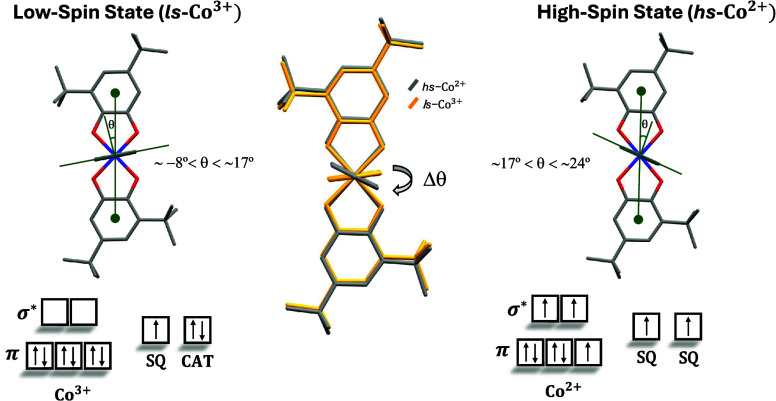

## Introduction

Electronic bistability is a property exhibited by certain compounds that can interconvert between distinct electronic spin states. Because of their responsive magnetic and electronic properties, these systems have long been proposed for technological applications such as molecular spintronics for data storage^1,2^ and chemosensing.^3^ Bistability in these compounds can arise from various phenomena, including mixed valence (MV),^4^ spin crossover (SCO),^5^ and valence tautomerism (VT).^6,7^ In contrast to classical spin crossover, VT couples a single-site spin transition at the metal center with intramolecular metal–ligand charge transfer, giving rise to distinct redox isomers. Valence-tautomeric interconversion (VTI) can therefore be viewed as an entropy-driven, often reversible redistribution of intramolecular valence electrons that triggers a single-site spin-state change accompanied by metal–ligand charge transfer, producing switchable redox isomers with different spectroscopic and magnetic signatures. VTI can occur both in solution and in the solid state without disrupting the crystalline lattice. It is well established that in valence-tautomeric compounds, interconversion between spin states correlates with a change in the oxidation state of the metal center, which is often accompanied by structural changes, including variations in metal–ligand bond distances and in the bond lengths within the coordinated ligands.^7,8^ VTI can also be induced and controlled by a variety of external stimuli, including temperature,^9–12^ pressure,^13,14^ magnetic- and electric-field changes,^15,16^ irradiation with visible light,^11,12,17,18^ and soft and hard X-rays.^13,19,20^ Temperature-dependent VTI can occur as either a first-order (abrupt) or second-order (gradual) transition over a wide range of characteristic transition temperatures.^9–11,18^ Additionally, VTI in the solid state is influenced by packing effects, which may enable, inhibit, or modulate the interconversion between redox isomers.^10,21^

Since its first application to the [Co­(dioxolene)_2_(N_x_L)] (N_*X*_L = nitrogen based ancillary ligand, *X* = number of nitrogen atoms at the ligands) family in 1997,^22^ density functional theory (DFT) calculations have been extensively used to rationalize experimental observations by quantifying spin-state energetics, charge redistribution, and metal–ligand covalency in VT complexes. More recently, DFT has evolved from an interpretive to a predictive approach, enabling the anticipation of VT behavior in diverse bidentate cobalt-o-dioxolene complexes.^23–25^ Despite these advances, modeling VTI remains challenging, as some functionals tend to overstabilize specific spin states, thereby introducing bias into the calculations.^21,23,26^

The present study focuses on understanding solid-state VTI in a cobalt complex coordinated by redox-active o-dioxolene ligands, either in the semiquinone (SQ) form (3,5-di-*tert*-butylsemiquinonate) or the catecholate (CAT) form (3,5-di-*tert*-butylcatecholate), with pyridine (Py) as an ancillary ligand, denoted as [Co­(dioxolene)_2_(Py)_2_]. Figure 1 schematically depicts the general interconversion mechanism between the different spin and redox states in Co­(dioxolene) complexes bearing pyridyl-like ancillary ligands (PyL). It illustrates VTI between a low-spin [Co^3+^(CAT)­(SQ)­(PyL)_2_] with *S* = 1/2 isomer (hereafter *ls*-Co^3+^) at lower temperatures and a high-spin [Co^2+^(SQ)_2_(PyL)_2_] with *S* = 5/2 isomer (hereafter *hs*-Co^2+^) at higher temperatures. In the CAT form, the aromatic ring forms single bonds with both oxygen atoms, each of which possesses two lone electron pairs and no unpaired spins. Upon conversion to the SQ form, one oxygen atom donates an electron to the metal center, forms a double bond with the aromatic ring, and retains only one lone-pair electron. This electronic redistribution generates an unpaired electron in the semiquinone. Because of its resonant character, the position of the double bond is delocalized over both oxygen atoms, resulting in the corresponding delocalization of the unpaired spin density.

**Figure 1:**
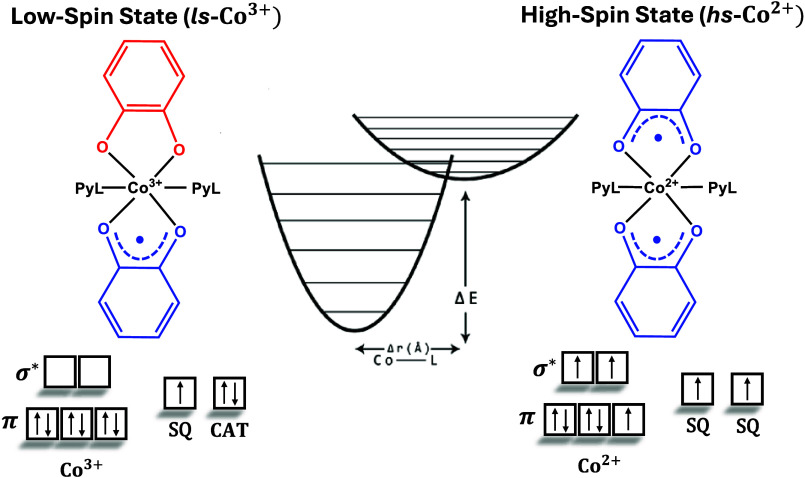
Scheme representing the interconversion from *ls*-Co^3+^ to *hs*-Co^2+^. The dioxolene in catecholate form (red) displays exclusively single bonds between the oxygen atoms and the aromatic ring. In the semiquinone form (blue), a resonant double bond is established, represented by the dashed lines. The electronic distribution changes during the interconversion between the *ls*-Co^3+^ and *hs*-Co^2+^ states, both of which are stable under specific conditions.

As indicated in Figure 1, VTI in cobalt-o-dioxolene complexes is accompanied by reversible changes in the Co–L bond lengths (where L = O or N atoms from the dioxolene and ancillary ligands in the first coordination sphere). These quasi-isotropic variations in Co–L bond lengths are directly correlated with the oxidation state of cobalt,^7^ while the bond distances within the quinone ligand can be used to calculate the empirical metrical oxidation state (MOS), which represents the apparent oxidation state of the ligand.^8^ Previous studies have shown that both Co–L bond lengths and the MOS method can reliably determine the oxidation state of the system, thereby enabling estimation of the relative redox-isomer (*ls*-Co^3+^/*hs*-Co^2+^) population at a given temperature.^12^ A detailed comparison of [Co­(dioxolene)_2_(Py)_2_] complexes at different temperatures reveals that the plane defined by the Py ligands undergoes pronounced torsion in the *hs*-Co^2+^ state relative to the *ls*-Co^3+^ state, as shown in Figure 2, where θ is defined as the angle between the vector normal to the pyridine-ligand plane and the vector connecting the centroids of the two dioxolene rings. Similar ligand torsions have been observed in other systems exhibiting VT and SCO, including time-dependent torsions of bipyridine in Fe^2+^-bipyridine complexes^27,28^ and torsional deformations within the coordination planes of [Co­(dioxolene)­(N_4_L)]^+^ complexes.^24,25^

**Figure 2:**
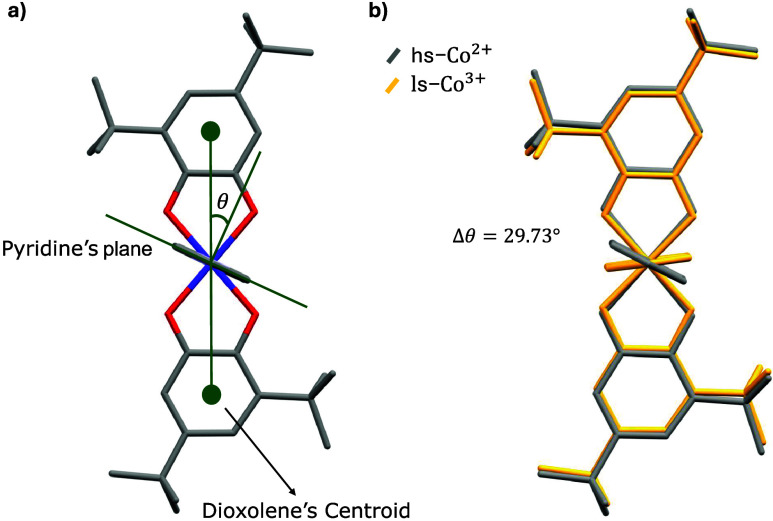
(a) Angle θ between the vector connecting the centroids of the dioxolene rings and the vector normal to the pyridine-ligand plane. Co atoms are depicted in purple, O atoms in red, and C atoms in gray. H atoms were omitted for clarity. (b) The pyridine planes differ markedly in the *hs*-Co^2+^ form relative to the *ls*-Co^3+^ state, as shown by the superposition of the molecules.

The complex [Co­(dioxolene)_2_(Py)_2_] is known to crystallize in several solvated forms, with the presence or absence of VTI being strongly governed by solvation effects, as demonstrated by Mulyana et al.^9^ and Schmidt et al.^11^ Variable-temperature magnetic-susceptibility studies show that the 1:2 complex/solvent acetonitrile solvate, [Co­(dioxolene)_2_(Py)_2_]/acetonitrile (**C-1:2A**), exhibits a temperature-dependent, partially reversible VTI response over successive heating–cooling cycles between 25 and 370 K, whereas the fully air-dried solid obtained from the same 1:2 complex/solvent ratio (**C-1:0A**) shows no evidence of VTI between 20 and 350 K.^9^ No structural comparison between the corresponding redox forms was carried out in that study. Similarly, variable-temperature magnetic-susceptibility data indicate that the 1:0.5 toluene solvate, [Co­(dioxolene)_2_(Py)_2_]/toluene (**C-1:0.5T**), does not exhibit VTI from 20 to 300 K,^11^ and single-crystal X-ray diffraction (SCXRD) analysis shows that the 1:1.67 hexane solvate, [Co­(dioxolene)_2_(Py)_2_]/hexane (**C-1:1.67H**), is also nontautomeric in the 130–300 K range.^9^ In contrast, the variable-temperature magnetic susceptibility and SCXRD results for the 2:1 pyridine solvate, [Co­(dioxolene)_2_(Py)_2_]/pyridine (**C-2:1P**), reveal VTI in only one of the two crystallographically independent [Co­(dioxolene)_2_(Py)_2_] asymmetric units.^9^ Notably, in **C-2:1P**, the Py ligands of the nontautomeric units engage in π–π stacking with the pyridine solvate molecules, whereas in **C-1:2A**, the Py ligands also form π–π stacking interactions, a feature reputed to suppress VTI at that site.^9^

Previous work has shown that packing and solvation modulate VTI in [Co­(dioxolene)_2_(PyL)_2_] complexes, but a direct link among pyridyl torsion, solvation, Co–L distances (redox state), and VT energetics remains unclear. Notably, the ancillary Py ligands were not originally expected to play a direct role in the VT process, as they are only weakly perturbed by changes in electronic distribution,^23^ and the presence or absence of VTI in [Co­(dioxolene)_2_(Py)_2_] solvates had so far been rationalized mainly in terms of simple steric crowding around the Py ligands.

Here, we combine experimental and computational analyses to elucidate how VT interconversion correlates with molecular structure in cobalt-o-dioxolene systems, thereby identifying the key geometric factors that govern valence tautomerism and establishing a clear mechanism for geometric–electronic coupling in this family behavior. As part of this effort, we carried out a detailed reinvestigation of the **C-2:1P** structures over heating and cooling cycles between 100 and 290 K. In addition, we report the synthesis and structural characterization of the 1:2 pyridine solvate, [Co­(dioxolene)_2_(Py)_2_]·pyridine (**C-1:2P**). In this crystal, π–π stacking between the Py ligands in neighboring molecules locks the complex in the *ls*-Co^3+^ state from 100 K up to 273 K, thereby suppressing VTI.

DFT geometry optimizations based on the experimentally determined atomic coordinates within the **C-2:1P** crystal show that stabilization of the *hs*-Co^2+^ state is systematically associated with larger θ torsion angles of the pyridyl ligand plane, whereas stabilization of the *ls*-Co^3+^ state corresponds to smaller torsions, in full agreement with the experimental observations (Figure 2). In addition, the VTI was evaluated from the distribution of spin polarization, as revealed by DFT-calculated spin-density isosurfaces.

## Experimental Section

### Complex Synthesis and Crystallization

All chemicals used for synthesis and analysis were of analytical grade and were used without further purification. The synthesis of the [Co­(dioxolene)_2_(Py)_2_] complex (dioxolene = 3,5-di-*tert*-butyl-o-semiquinonate/3,5-di-*tert*-butyl-o-catecholate; Py = pyridine) was previously reported by Schmidt et al.^11^ and was reproduced here.

[Co­(dioxolene)_2_(Py)_2_] crystals with a 2:1 complex/pyridine solvent ratio (**C-2:1P**) were prepared by dissolving 0.1 g of [Co­(dioxolene)_2_(Py)_2_] powder in 15 mL of hexane. The solution was stirred and heated to 60 °C and then left at room temperature for 15 min, after which 2 μL of pyridine was added. The final solution was left partially covered at room temperature to allow slow evaporation of hexane overnight, yielding dark-green needle-like crystals suitable for SCXRD experiments.

[Co­(dioxolene)_2_(Py)_2_] crystals with a 1:2 complex/pyridine solvent ratio (**C-1:2P**) were prepared by dissolving 0.1 g of [Co­(dioxolene)_2_(Py)_2_] powder in 30 mL of hexane. The solution was stirred and heated to 60 °C and then left at room temperature for 15 min, after which 3 mL of pyridine was added. The final solution was left partially covered at room temperature to allow slow evaporation of hexane overnight, yielding dark-green needle-like crystals suitable for SCXRD experiments.

### Single-Crystal X-ray Diffraction Data and Structure Solution

Samples of **C-2:1P** were examined by SCXRD on a Rigaku Oxford Diffraction Gemini diffractometer using MoKα radiation (λ = 0.71 Å), whereas **C-1:2P** data were collected at the Manacá beamline of the Brazilian Sirius Light Source using 18.5 keV X-rays (λ = 0.6702 Å).^29^ In both cases, crystals were manually mounted on cryo-loops and subjected to sequential temperature-dependent measurements on the same specimen. For **C-2:1P**, data were collected between 100 and 290 K, whereas for **C-1:2P**, at 100 and 273 K. SCXRD images were recorded using a CCD detector for **C-2:1P** and a PILATUS 2M detector for **C-1:2P**. SCXRD data for **C-2:1P** were processed with CrysAlis,^30^ whereas data for **C-1:2P** were processed with XDS.^31^ Both structures were solved with SHELXT^32^ and refined using SHELXL.^33^

The **C-2:1P** crystal shows *P*2_1_/*c* symmetry and contains two symmetry-independent Co­(dioxolene)­(Py) moieties and half of a pyridine solvate molecule in the asymmetric unit (AU) (colored atoms in Figure 3a). Application of inversion symmetry to the AU atoms generates two full [Co­(dioxolene)_2_(Py)_2_] complexes, with the cobalt atoms occupying Wyckoff positions *a* and *d*. Only one of these, designated molecule B in Figure 3a, interacts with a pyridine solvent molecule, as previously reported.^9^ Despite the high quality of the SCXRD data, the nitrogen position in the pyridine solvate could not be unambiguously determined and was therefore replaced by a carbon atom during refinement. The **C-1:2P** crystal adopts *P*2_1_/*c* symmetry and contains one [Co­(dioxolene)_2_(Py)_2_] moiety together with one pyridine molecule in the asymmetric unit (AU) (colored atoms in Figure 3b). The nitrogen position of the pyridine solvate could be identified in the 100 K data set; however, at 273 K, it was replaced by carbon because of solvent disorder, which prevented reliable determination of its position. Application of inversion symmetry to the AU atoms generates a second molecule that interacts with this complex through π–π stacking.

**Figure 3:**
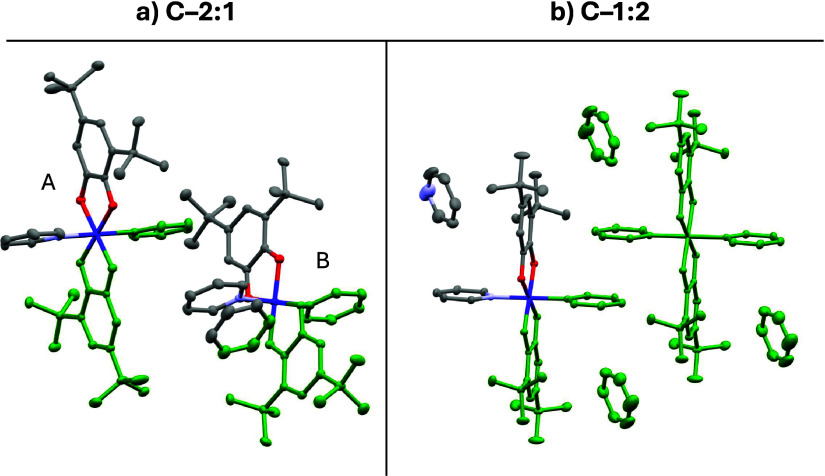
(a) **C-2:1P**, with one solvent molecule per two [Co­(dioxolene)_2_(Py)_2_] complex molecules, with symmetry-independent molecules A and B indicated; (b) **C-1:2P**, with two solvent molecules per complex unit. Carbon atoms are depicted in gray, oxygen in red, nitrogen in blue, and cobalt in purple. Hydrogen atoms are omitted for clarity. The pyridine nitrogen atom in **C-2:1P** was replaced by a carbon atom because its position in the ring could not be reliably determined. Symmetry-equivalent parts are depicted in green. Both figures were obtained for 100 K.

For both **C-2:1P** and **C-1:2P**, hydrogen atoms were located in Fourier difference maps and refined as riding atoms (C–H and N–H = 0.97 Å; Uiso­(H) = 1.2 Ueq­(C or N) for methylene, aromatic, and carbon-bound hydrogens), and all non-hydrogen atoms were refined with anisotropic displacement parameters. Mercury^34^ was used for crystal-structure visualization, analysis, and figure preparation. Crystal data and refinement indicators are summarized in the Supporting Information (SI) Tables S1–S3.

### DFT Calculations

DFT calculations were performed using the SIESTA software package (version 4.1.5).^35^ The magnetic-susceptibility data reported elsewhere^9^ were used as the experimental reference for functional selection because, through Curie’s law for solids, magnetic susceptibility is related to the total spin moment (*J*), which can be obtained from DFT calculations. Previous studies have shown that the functionals B3LYP* (with 15% HF) and OPBE provide the best agreement with experimental energy gaps (Δ*E*) between the *ls*-Co^3+^ and *hs*-Co^2+^ spin states.^21,24,26,36,37^ However, some functionals tend to overstabilize one of the states. Different functionals are also known to produce small differences in bond distances,^26^ which is critical for these systems. The PBE-GGA^38^ and VDW^39^ functionals, for instance, tend to overestimate bond lengths, resulting in spin-moment oscillations that are inconsistent with the experimental measurements. The local density approximation (LDA)^40^ provided the best agreement with the experimental measurements successfully predicting the interconversion temperature (*T*_1/2_) and was therefore employed for geometry optimization of [Co­(dioxolene)_2_(Py)_2_], yielding consistent results. The calculations were performed with a DZP (double-ζ plus polarization) basis set, a 300 Ry real-space mesh, and an 8 × 8 × 4 Monkhorst–Pack k-point mesh. Furthermore, the geometries were relaxed until the force on every atom reached 0.04 eV/Å or less, and the convergence criterion for the electronic density matrix was set to 10^–4^. Spin-density isosurfaces were then calculated to show how spin polarization is distributed in real space, allowing the spin-state transition to be identified.

DFT calculations were performed on **C-1:2P** and **C-2:1P** using, as starting geometries, the crystal structures obtained by X-ray diffraction at 100 and 273 K for **C-1:2P** and over the 100–290 K range for **C-2:1P**. For **C-1:2P**, all calculations yielded the expected result: the complex in the asymmetric unit remained in the *ls*-Co^3+^ state at both temperatures. In contrast, for **C-2:1P**, geometry optimizations produced only negligible structural changes in the nontautomeric unit (molecule B in Figure 3), which remained *ls*-Co^3+^ in all cases, whereas the tautomeric unit (molecule A in Figure 3) adjusted its geometry to match the stable spin and redox state closest to the temperature at which the input structure was obtained.

Experimental data for **C-2:1P** show that the magnetic susceptibility increases monotonically upon heating, as indicated in Figure 4 (adapted from Mulyana et al.^9^). Magnetic susceptibility can be obtained using Curie’s law for solids:

$$\chi_{M}=\frac{\mu_{0}N_{A}\mu_{\mathrm{eff}}^{2}\mu_{B}^{2}}{3K_{B}T}$$

**Figure 4:**
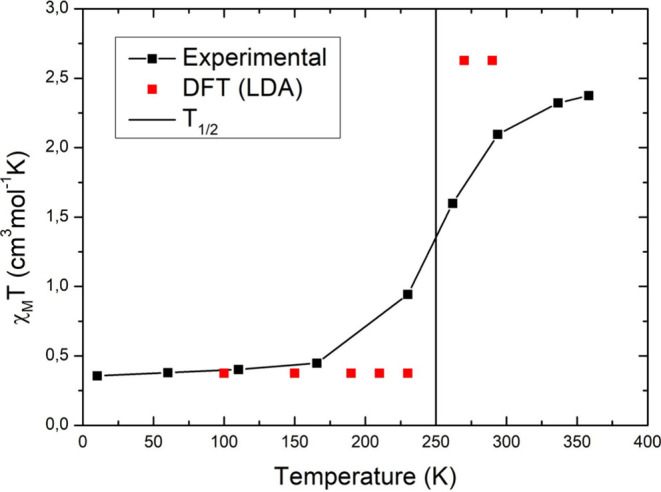
Comparison between the magnetic susceptibility for **C-2:1P** calculated with the LDA functional and the values obtained experimentally by Mulyana et al.^9^ For **C-2:1P**, *T*_1_/_2_, defined as the temperature at which half of the tautomeric units are in the *hs*-Co^2+^ state and the other half remain in the *ls*-Co^3+^ state, occurs at 250 K.

From this relation, the effective moment (μ_eff_) can be expressed as a function of magnetic susceptibility and temperature:

$$\mu_{\mathrm{eff}}=2.827\sqrt{\chi_{M}^{\mathrm{cgs}}T}$$

The total spin moment can be obtained from DFT calculations and is related to the effective moment by the relation:

$$\mu_{\mathrm{eff}}^{2}=J(J+1)g$$

Since each unit cell contains four complexes, the total spin moment per unit cell is given by the sum of the individual spin moments of each complex. Because SIESTA does not impose multiplicity as a constraint, magnetic susceptibility can be calculated and directly compared with experimental data. For the **C-2:1P** structure, LDA yielded the best agreement with experiment among the tested functionals, satisfactorily reproducing *T*_1_/_2_ ≈ 250 K, defined as the temperature at which half of the tautomeric units are in the *hs*-Co^2+^ state and the other half remain in the *ls*-Co^3+^ state (Figure 4). At this temperature, **C-2:1P** (A) presents Co–N bond lengths and torsion θ angles within 2.035(2) Å and 18.2(1)° of the experimental values (see Figures S6 and S7).

Even at temperatures at which the system reaches saturation of the magnetic susceptibility (>350 K), the transition to *hs*-Co^2+^ occurs in only two of the four molecules in the unit cell, namely, those with cobalt atoms at Wyckoff position *d*, whereas the other two, at Wyckoff position *a*, remain in the *ls*-Co^3+^ state. The increase in the effective magnetic moment observed in the experimental curve (Figure 4) for **C-2:1P** is gradual, resembling a second-order transition. This behavior indicates that the *ls*-Co^3+^ ↔ *hs*-Co^2+^ VTI does not occur simultaneously in all unit cells. Instead, at each temperature, the experimental magnetic susceptibility reflects the average response of complexes in different states throughout the crystal. DFT calculations, on the other hand, always seek points of stability, which eliminates average values from the theoretical results and gives the transition the abrupt behavior expected for a first-order transition. As shown in Figure 4, the calculated magnetic susceptibility for **C-2:1P** reaches stability in two redox-isomer forms below and above 250 K, respectively.

## Results and Discussion

DFT calculations were carried out on a single [Co­(dioxolene)_2_(Py)_2_] molecule using the atomic coordinates from **C-2:1P** SCXRD data collected at temperatures slightly below and above *T*_1/2_ for both the tautomeric and nontautomeric units (molecules A and B in Figure 3, respectively). At 230 K, geometry optimizations show that both the torsion angle θ and the Co–L (L = N, O) bond lengths change in the tautomeric unit, converging to values comparable to those of the nontautomeric unit (Figure 5), i.e., values consistent with a *ls*-Co^3+^ redox isomer.

**Figure 5:**
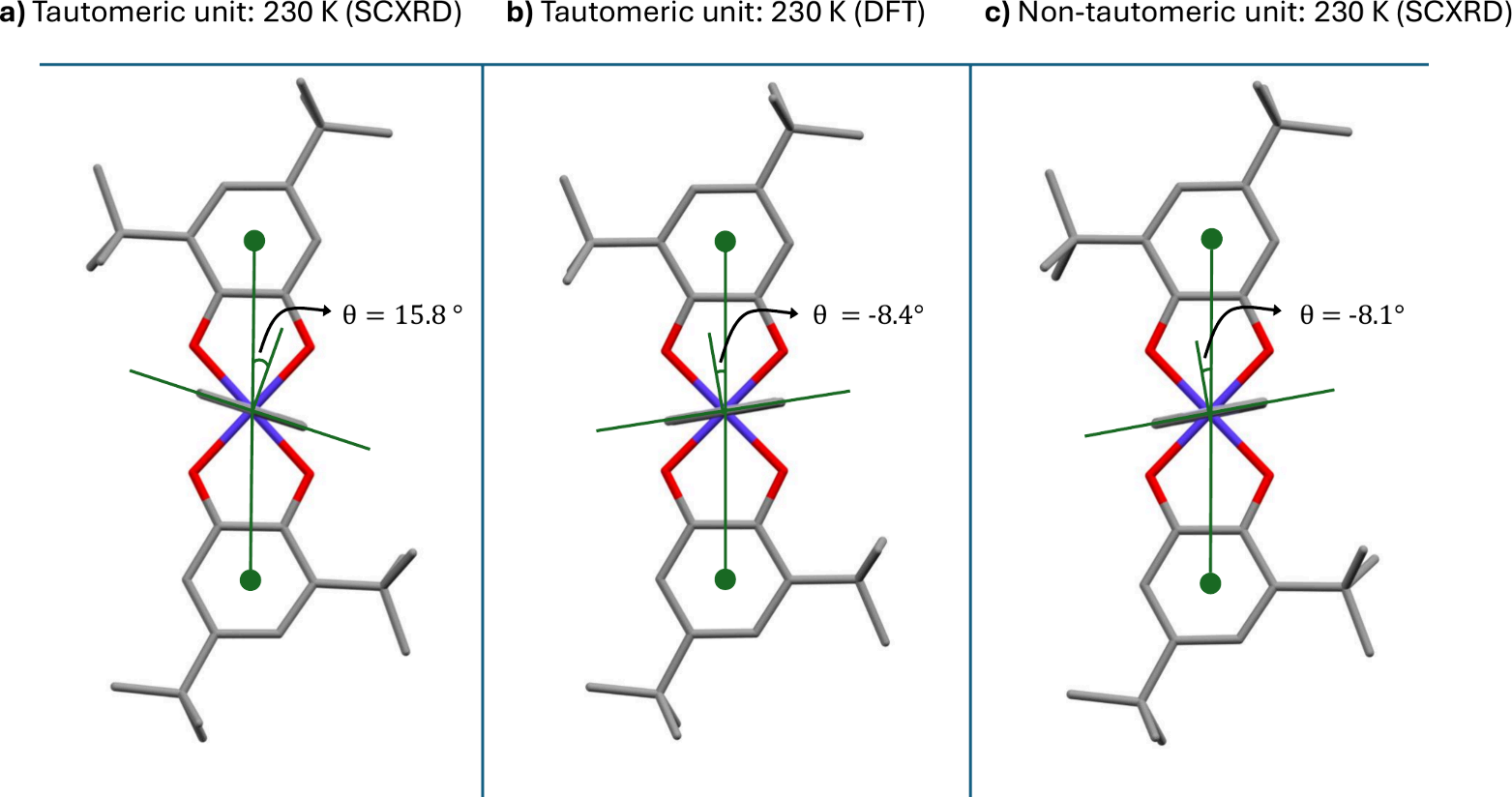
The tautomeric unit at 230 K (*ls*-Co^3+^), with a torsion angle θ of 15.8° (a), undergoes a significant change in this angle after atomic coordinate optimization via DFT calculations (b), becoming similar to the nontautomeric unit (c). Co atoms are depicted in purple, O atoms in red, and C atoms in gray. H atoms were omitted for clarity.

The same type of calculation was performed using the atomic coordinates obtained above *T*_1/2_, at 270 K. In this case, both the Co–L bond distances and the ligand torsion angle θ of the tautomeric unit remained almost unchanged after geometry optimization, i.e., consistent with an *hs*-Co^2+^ redox isomer (Figure 6).

**Figure 6:**
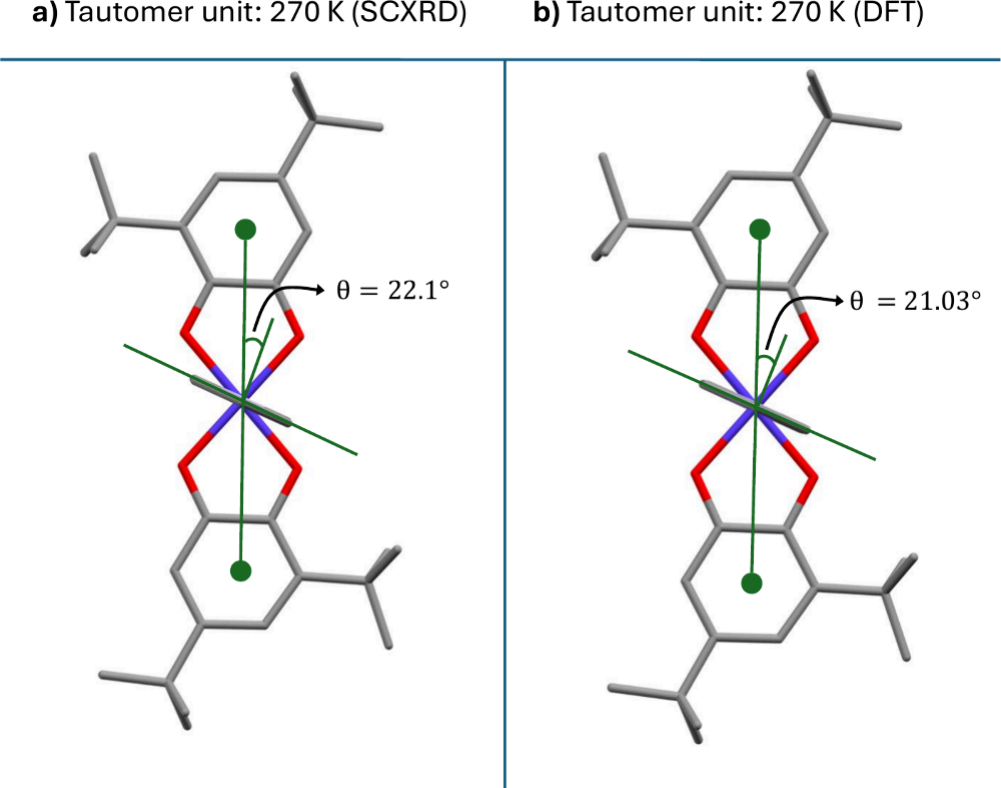
The tautomeric unit of **C-2:1P** at 270 K (*hs*-Co^2+^), with a torsion angle θ of 21.1° (a), remains almost unaffected after atomic-coordinate optimization by DFT (b), showing that the stabilization of the *hs*-Co^2+^ state is correlated with the torsion of the pyridine ligand. Co atoms are depicted in purple, O atoms in red, and C atoms in gray. H atoms were omitted for clarity.

The results in Figures 5 and 6 indicate that the pyridine torsion angle influences the occurrence of VTI in the **C-2:1P** complex. To assess whether this behavior is a general feature of Co­(dioxolene) complexes or is specific to [Co­(dioxolene)_2_(Py)_2_], we carried out a survey using the CSD tool ConQuest.^41^ This search focused on *trans*-[Co­(dioxolene)_2_(PyL)_2_] molecules, i.e., Co centers octahedrally coordinated by the oxygen atoms of two redox-active ligands and the nitrogen atoms of monodentate pyridyl-like ligands (PyL), yielding a total of 78 structures in the CSD. Among these, five were excluded from the analysis because their dioxolene ligands, pyridyl ligands, or both were not coplanar, making discussion of rotation inapplicable (see the SI, Table S4). For all the remaining structures, the correlation between the average Co–N distances and the torsion angle θ between the vector connecting the dioxolene ring centroids and the vector normal to the pyridyl-like ligand plane (see Figure 2) shows two linear regimes, as indicated in Figure 7. The Co–N bond distances remain virtually constant at ∼1.95 Å as the torsion angle θ increases up to a critical value of approximately 16.5°. This bond length is characteristic of *ls*-Co^3+^. Beyond this critical angle, a linear regime emerges in which the Co–N distance increases from ∼1.95 Å to ∼2.20 Å, typical of *hs*-Co^2+^, while the torsion angle expands from ∼16.5° to nearly 30°.

**Figure 7:**
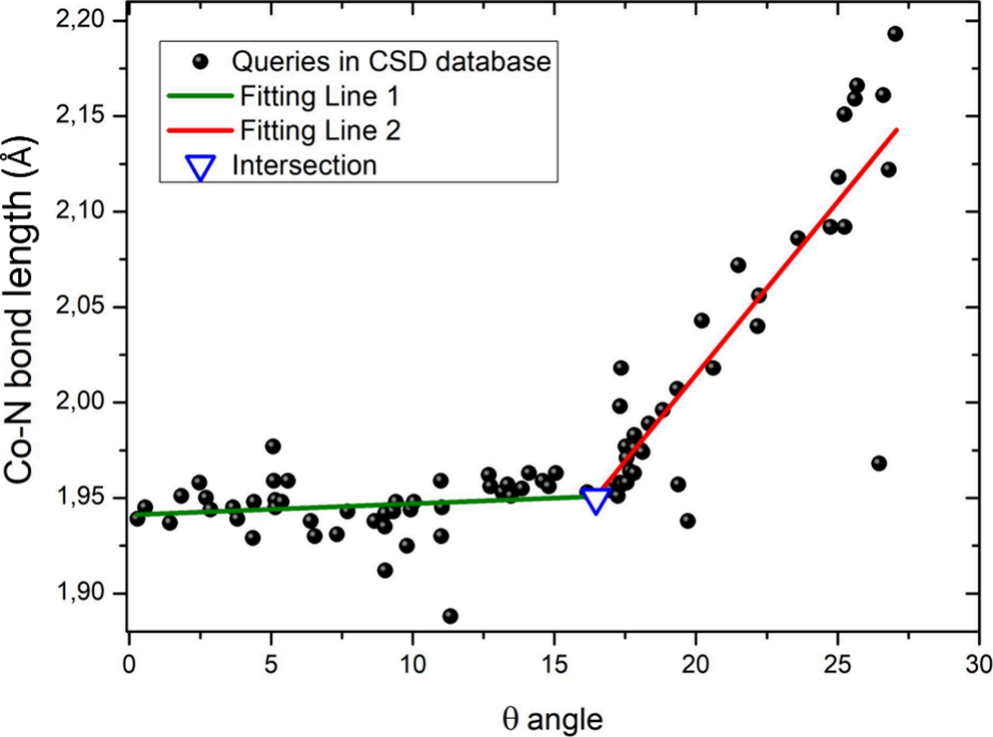
Average Co–N distances as a function of the angle between the PyL planes and the Co-dioxolene vectors for all surveyed *trans*-[Co­(dioxolene)_2_(PyL)_2_] structures.

The outlier highlighted in Figure 7 corresponds to the [Co­(dioxolene)_2_(OMe-Py)_2_] structure (CSD code: YUWYIZ),^17^ which exhibits short contacts between the OMe-Py ligands and neighboring molecules (see the SI, Figure S5). In this structure, pyridine C–H groups are attracted to the oxygen atoms of the OMe-Py groups bound to pyridines in adjacent complexes. Although this interaction causes the pyridine ring to rotate, it is not related to valence tautomerism. Instead, it results from a purely intermolecular interaction and does not depend on the redox states of cobalt or dioxolene.

It is worth emphasizing that Co–N bond distances in the range of ∼1.94–2.20 Å indicate partial conversion from the *ls*-Co^3+^ state toward the *hs*-Co^2+^ state.^7,12,20^ Thus, the correlation between Co–N bond length and θ in the surveyed structures highlights the key role of this torsion in understanding VTI, and a detailed picture of this feature in [Co­(dioxolene)_2_(Py)_2_] may lead to a more general description applicable to related complexes.

The calculated spin-density isosurfaces for the [Co­(dioxolene)_2_(Py)_2_] unit, shown in Figure 8a (*ls*-Co^3+^) and Figure 8b (*hs*-Co^2+^), reveal clear differences between the spin-up and spin-down charge distributions. As shown in Figure 8, the spin density in the *hs*-Co^2+^ state is significantly greater than that in the *ls*-Co^3+^ state, as indicated by the more extended yellow isosurfaces, consistent with the increase in the number of unpaired electrons from 1 (*ls*-Co^3+^) to 5 (*hs*-Co^2+^) upon VTI. The isosurfaces show that these unpaired electrons are mainly localized on the cobalt and oxygen atoms. In particular, all four oxygen atoms display appreciable spin density, in line with the delocalization of the C–O double bond among them, as schematically depicted in Figure 1.

**Figure 8:**
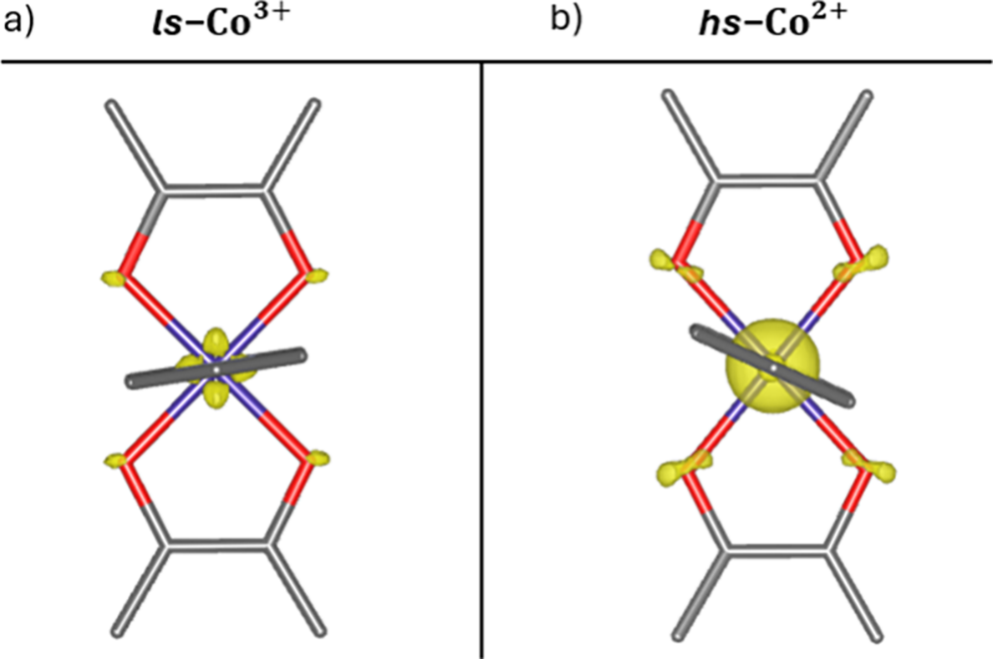
Calculated isosurfaces (yellow), obtained with the LDA functional, of the difference between the spin-up and spin-down charge densities in real space around [Co­(dioxolene)_2_(Py)_2_]. The calculations were performed after geometry optimization of the [Co­(dioxolene)_2_(Py)_2_] moieties in the **C-2:1P** structure obtained by X-ray diffraction (a) below *T*_1/2_ and (b) above *T*_1/2_. Both isosurfaces were set at a level of 0.047 e/Å^3^. Co atoms are depicted in purple, O atoms in red, and C atoms in gray. H atoms were omitted for clarity.

Analysis of the projected density of states (PDOS) for the oxygen atoms of the [Co­(dioxolene)_2_(Py)_2_] unit confirms an increase in the spin polarization between the two states. In the *ls*-Co^3+^ state, the spin difference, obtained by integrating the spin-up minus spin-down projected states, is similar across all four oxygen atoms. In the *hs*-Co^2+^ state (290 K), however, the spin density increases on all four oxygens, reaching particularly high values for the O2/O3 pair (Figure 9). To further probe the spin distribution, Mulliken atomic population analysis was performed for all single-molecule calculations, confirming enhanced spin polarization on the same O2/O3 pair of oxygen atoms.

**Figure 9:**
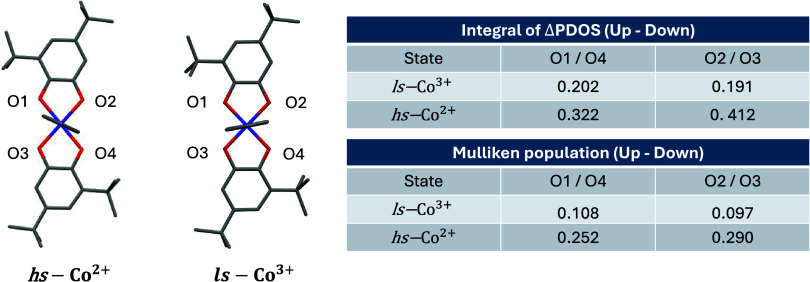
Integrated difference between spin-up and spin-down projected density of states, as well as the spin difference obtained from Mulliken analysis, indicates that spin polarization is significantly greater on the O2/O3 atoms than on O1/O4 in the *hs*-Co^2+^ state. In contrast, spin polarization is significantly lower in the *ls*-Co^3+^ state, and the oxygen atoms become much more similar.

In the *ls*-Co^3+^ state, a C=O double bond is delocalized over all four oxygen atoms, rendering them nearly equivalent. As a result, their interactions with the pyridine C–H groups are more uniform and do not induce torsion of the Py plane. In contrast, in the *hs*-Co^2+^ state, the unpaired spin and the C=O double bond in each semiquinone are more strongly delocalized over the O2 and O3 atoms. Consequently, O1 and O4 retain predominantly two lone electron pairs each, which appear to interact with the pyridine C–H groups and drive the observed torsion of the pyridine plane. This may help explain why π–π stacking prevents the VTI, as observed in **C-1:2P**. The steric hindrance associated with π–π stacking interactions between pyridine molecules (whether from neighboring complexes or pyridine solvate molecules) appears to prevent both the expansion in molecular volume that must accompany the VT transition—as suggested by Mulyana et al.^9^—and the delocalization of electronic density over O2 and O3 that favors stabilization of the *hs*-Co^2+^ state. Note that in the molecule exhibiting π–π stacking in **C-2:1P** (*ls*-Co^3+^ over the entire temperature range investigated), the electron density delocalization over O1 and O4 observed in the VT isomer does not occur, as indicated by the DPDOS and Mulliken population shown in table associated with Figure 9. It is important to stress, however, the resonant nature of both the double bond and the unpaired spin: the calculations point to a preferred configuration that stabilizes specific electrostatic interactions in the *hs*-Co^2+^ state (Figure 10), rather than a single rigid bonding pattern.

**Figure 10:**
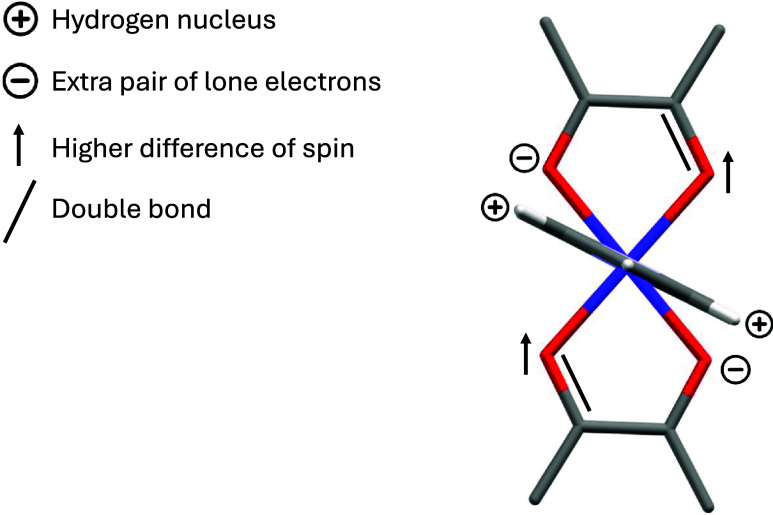
Schematic illustration of the preferential configuration in *hs*-Co^2+^, highlighting the electrostatic interactions associated with pyridine ligand torsion.

Another noteworthy insight into the role of pyridine orientation arises from calculations initiated with the torsion angle rotated in the opposite direction (Figure 11). These results show that the oxygen atoms displaying the largest spin-density differences depend on the torsion angle: the oxygens retaining predominantly single-bond character (and therefore smaller spin-density differences) are consistently those located closer to the pyridine C–H groups. This suggests that the preferred location of the C=O double bond is strongly influenced by the Py ligand-plane configuration, such that inverting the torsion reverses the pattern of spin polarization over the four oxygen atoms.

**Figure 11:**
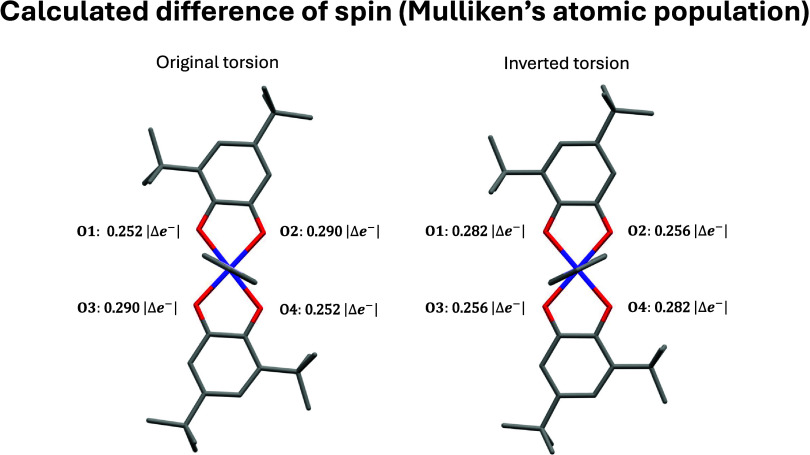
Difference of spin for each oxygen atom, obtained from Mulliken population analysis. The inversion of the pyridine ligand’s plane also inverts the oxygen atoms, exhibiting the highest spin polarization.

## Concluding Remarks

The occurrence of valence tautomeric interconversion (VTI) in [Co­(dioxolene)_2_(Py)_2_] is shown to depend critically on crystal packing, solvation, and their impact on the steric environment of the pyridine ligands. Analysis of our single-crystal X-ray diffraction (SCXRD) data collected on solvated [Co­(dioxolene)_2_(Py)_2_] crystals at various temperatures reveals a consistent correlation between the torsion angle of the pyridine ancillary ligands and the electronic and spin states, demonstrating that pyridine torsion is a key structural parameter for *ls*-Co^3+^ ↔ *hs*-Co^2+^ interconversion. The observation of analogous structure–property relationships in other cobalt-o-dioxolene complexes bearing PyL ancillary ligands, in which VTI is induced by a variety of external stimuli, further underscores the general relevance of this torsional degree of freedom.

DFT calculations additionally provided a microscopic rationale for this behavior. In the catecholate *ls*-Co^3+^ form of [Co­(dioxolene)_2_(Py)_2_], each O atom bears two lone pairs and forms two Co–O single bonds. Upon conversion to the semiquinone *hs*-Co^2+^ form, one lone electron is engaged in a C=O π bond, while the other is donated to the metal center, leaving that oxygen with a single lone pair. Although spin density and double-bond character are delocalized over all oxygen atoms, DFT calculations indicate the existence of a preferred configuration. Crucially, localization on a specific O atom reduces its ability to engage in stabilizing interactions because of the loss of a lone pair. In contrast, O atoms retaining two lone pairs can interact more strongly with the partial positive charge of nearby hydrogen nuclei, promoting torsion of the pyridine ring to optimize these electrostatic contacts. When this torsional motion is sterically constrained, such charge stabilization is hindered, effectively increasing the energy gap between spin states and disfavoring VTI.

Overall, our results show that although PyL ligand torsion is not sufficient on its own to induce VTI, constraining this degree of freedom can completely suppress it. However, the strength of ligand-based intramolecular interactions in [Co­(dioxolene)_2_(PyL)_2_] complexes can be indirectly modulated by solvation and crystal packing, allowing these interactions to act as structural triggers for VT.

Taken together, our findings define ligand-torsion-based design rules for controlling bistability in spin- and valence-switchable coordination compounds and guiding their implementation in practical applications.

## Supplementary Material



## Data Availability

Crystallographic data supporting the findings of this study are available from the Cambridge Crystallographic Data Centre (CCDC) under deposition numbers 2535400–2535409.

## Notes

Crystal data and refinement indicators, detailed descriptions of the ConQuest search, and DFT calculations (PDF)
