# Supplementary material for: Uncovering Correlations between Structure and Valence Tautomerism in Cobalt-o-Dioxolene Crystals
Source: J Phys Chem C Nanomater Interfaces. 2026 Jul 8;130(29):10398–407. doi: 10.1021/acs.jpcc.6c02263 (PMC13403308; doi:10.1021/acs.jpcc.6c02263)
Supplement: Supplementary file 1 [file jp6c02263_si_001.pdf]

Uncovering Correlations Between Structure and Valence Tautomerism in Cobalt-o-Dioxolene Crystals

Marcelo Francis Fernandes Alecrim[a], Ludmila Leroy[a,b], Lucas Gustavo Gonçalves Pimenta[a], Lucas Olímpio Michel Machado[a], Majed Chergui[b,c], Simone Silva Alexandre[a] and Carlos Basílio Pinheiro[a]*

[a]Universidade Federal de Minas Gerais, Physics Department, Av.Antônio Carlos, 6627, 31270-901, Belo Horizonte, Minas Gerais, Brazil

[b]Lab. of Ultrafast Spectroscopy ISIC, FSB-BSP, École Polytechnique Fédérale de Lausanne, CH-1015 Lausanne, Switzerland

[c]Elettra-Sincrotrone Trieste, SS 14, km 163.5, 34149 Basovizza, Trieste, Italy

Supplementary Information

Crystallography data

Table S1: Crystal data and refinement indicators for **C-1:2P** at 100 K and 273 K.

**Identification code**	**CoSqPy1Py2-100 K**	**CoSqPy1Py2-273 K**
Empirical formula	C48 H60 Co N4 O4	C50 H62 Co N2 O4
Formula weight	815.93	813.94
Temperature (K)	100(2)	273(2)
Wavelength (Å)	0.6702	0.6702
Crystal system	Monoclinic	Monoclinic
Space group	P 21/c	P 21/c
Unit cell dimensions	*a* = 8.822(3) Å	*a* = 9.0030(9) Å
	b = 18.7630(17) Å	*b* = 18.9580(8) Å
	c = 13.2370(17) Å	*c* = 13.3120(7) Å
	β= 96.725(5)°	β= 96.600(5)°
Volume (Å^3)	2176.0(7)	2257.0(3)
Z	2	2
Density (calculated) (Mg/m^3)	1.245	1.198
Absorption coefficient (mm^{-1})	0.380	0.366
F(000)	870	870
Crystal size (mm^3)	0.100 x 0.025 x 0.025	0.100 x 0.025 x 0.025
Theta range for data collection	1.784 to 30.091°.	1.771 to 30.092°.
Index ranges	-12<=h<=12, -27<=k<=26, -19<=l<=19	-12<=h<=12, -28<=k<=28, -19<=l<=19
Reflections collected	37028	37737
Independent reflections	6300 [R(int) = 0.0348]	6521 [R(int) = 0.0473]

Completeness to θ = 23.7°	99.9 %	99.8 %
Refinement method	Full-matrix least-squares on F2	Full-matrix least-squares on F2
Data / restraints / parameters	6300 / 0 / 266	6521 / 36 / 328
Goodness-of-fit on F^2	1.116	1.078
Final R indices [I>2σ(I)]	R1 = 0.0369, wR2 = 0.0996	R1 = 0.0472, wR2 = 0.1243
R indices (all data)	R1 = 0.0376, wR2 = 0.1004	R1 = 0.0490, wR2 = 0.1274
Extinction coefficient	0.0203(13)	0.150(5)
Largest diff. peak and hole (e.Å^{-3})	0.543 and -0.597	0.480 and -0.498
**Selected Bond Distances**		
Co1 - O2 (Å)	1.8797(8)	1.8815(8)
Co1 - O1 (Å)	1.8860(8)	1.8855(10)
Co1 - N1 (Å)	1.9421(9)	1.9457(10)
O2 - C1 (Å)	1.3245(11)	1.3218(13)
O1 - C6 (Å)	1.3247(11)	1.3223(12)

Table S2: Crystal data and refinement indicators for **C-2:1P** from 100 K to 210 K.

**Identification code**	**CoSqPy2Py1-100 K**	**CoSqPy2Py1-150 K**	**CoSqPy2Py1-190 K**	**CoSqPy2Py1-210 K**
Empirical formula	C41 H53 Co N2 O4	C41 H53 Co N2 O4	C41 H53 Co N2 O4	C41 H53 Co N2 O4
Formula weight	696.78	696.78	696.78	696.78
Temperature (K)	100(2)	150(2)	190(2)	210(2)
Wavelength (Å)	1.54184	1.54184	1.54184	1.54184
Crystal system	Monoclinic	Monoclinic	Monoclinic	Monoclinic
Space group	P 21/c	P 21/c	P 21/c	P 21/c
Unit cell dimensions	*a* = 16.6280(2) Å	*a* = 16.6813(2) Å	*a* = 16.71953(19) Å	*a* = 16.73620(19) Å
	b = 18.6793(2) Å	*b* = 18.7213(2) Å	*b* = 18.7728(2) Å	*b* = 18.8135(2) Å
	c = 12.49673(17) Å	*c* = 12.52166(16) Å	*c* = 12.54467(15) Å	*c* = 12.55638(15) Å
	β= 108.3302(14)°	β= 108.2360(13)°	β= 108.1154(13)°.	β= 108.0045(12)°
Volume (Å^3)	3684.52(9)	3714.07(8)	3742.26(8)	3759.99(8)
Z	4	4	4	4
Density (calculated) (Mg/m^3)	1.256	1.246	1.237	1.231
Absorption coefficient (mm^{-1})	3.982	3.951	3.921	3.902
F(000)	1488	1488	1488	1488
Crystal size (mm^3)	0.366 x 0.162 x 0.095	0.366 x 0.162 x 0.095	0.366 x 0.162 x 0.095	0.366 x 0.162 x 0.095
Theta range for data collection	3.666 to 65.558°.	3.655 to 65.510°.	3.644 to 65.501°.	3.637 to 65.531°.
Index ranges	-19<=h<=19, -20<=k<=21, -14<=l<=12	-19<=h<=19, -21<=k<=22, -14<=l<=14	-19<=h<=19, -21<=k<=22, -14<=l<=14	-19<=h<=19, -21<=k<=22, -14<=l<=14
Reflections collected	26442	32780	33048	33263
Independent reflections	6295 [R(int) = 0.0300]	6368 [R(int) = 0.0289]	6419 [R(int) = 0.0305]	6450 [R(int) = 0.0308]
Completeness to θ = 65.5°	99.0 %	99.5 %	99.6 %	99.5 %
Absorption correction	Analytical	Analytical	Analytical	Analytical
Max. and min. transmission	0.852 and 0.639	0.852 and 0.639	0.852 and 0.639	0.852 and 0.639
Refinement method	Full-matrix least-squares on F2	Full-matrix least-squares on F2	Full-matrix least-squares on F2	Full-matrix least-squares on F2
Data / restraints / parameters	6295 / 5 / 436	6368 / 5 / 436	6419 / 11 / 436	6450 / 11 / 436
Goodness-of-fit on F2	1.139	1.089	1.036	1.060
Final R indices [I>2s(I)]	R1 = 0.0380, wR2 = 0.0880	R1 = 0.0356, wR2 = 0.0848	R1 = 0.0346, wR2 = 0.0859	R1 = 0.0367, wR2 = 0.0925
R indices (all data)	R1 = 0.0435, wR2 = 0.0906	R1 = 0.0419, wR2 = 0.0882	R1 = 0.0436, wR2 = 0.0920	R1 = 0.0476, wR2 = 0.1000
Extinction coefficient	n/a	n/a	n/a	n/a
Largest diff. peak and hole (e.Å^{-3})	0.220 and -0.360	0.224 and -0.387	0.309 and -0.398	0.425 and -0.352
**Selected Bond Distances**				
Co1 - O1 (Å)	1.8886(13)	1.8893(12)	1.8925(11)	1.8996(12)
Co1 - O2 (Å)	1.8962(13)	1.8962(12)	1.9005(12)	1.9109(12)
Co1 - N1 (Å)	1.9504(16)	1.9525(15)	1.9600(14)	1.9719(16)
Co2 - O3 (Å)	1.8783(13)	1.8799(12)	1.8801(11)	1.8808(12)
Co2 - O4 (Å)	1.8853(13)	1.8838(12)	1.8818(11)	1.8816(12)
Co2 - N2 (Å)	1.9517(17)	1.9522(16)	1.9531(16)	1.9523(17)
O4 - C20 (Å)	1.328(2)	1.326(2)	1.328(2)	1.326(2)
O3 - C21 (Å)	1.327(2)	1.329(2)	1.328(2)	1.327(2)
O2 - C6 (Å)	1.331(2)	1.330(2)	1.328(2)	1.326(2)
O1 - C1 (Å)	1.331(2)	1.330(2)	1.326(2)	1.324(2)

Table S3: Crystal data and refinement indicators for **C-2:1P** from 230 K to 290 K.

**Identification code**	**CoSqPy2Py1-230 K**	**CoSqPy2Py1-250 K**	**CoSqPy2Py1-270 K**	**CoSqPy2Py1-290 K**
Empirical formula	C41 H53 Co N2 O4	C41 H53 Co N2 O4	C41 H53 Co N2 O4	C41 H53 Co N2 O4
Formula weight	696.78	696.78	696.78	696.78
Temperature (K)	230(2)	250(2)	270(2)	293(2)
Wavelength (Å)	1.54184	1.54184	1.54184	1.54184 Å
Crystal system	Monoclinic	Monoclinic	Monoclinic	Monoclinic
Space group	P 21/c	P 21/c	P 21/c	P 21/c
Unit cell dimensions	*a* = 16.7542(2) Å	*a* = 16.7746(2) Å	*a* = 16.7969(3) Å	*a* =16.815(5) Å
	b = 18.8641(2) Å	*b* = 18.9325(2) Å	*b* = 18.9977(3) Å	*b* =19.054(5) Å
	c =12.56997(16) Å	*c* =12.58275(18) Å	*c* =12.5889(2) Å	y =12.599(5) Å
	β= 107.8705(13)°.	β = 107.6898(15)°	β = 107.462(2)°.	β = 107.277(5)°.
Volume (Å^3)	3781.11(9)	3807.14(9)	3832.02(12)	3854(2)
Z	4	4	4	4
Density (calculated) (Mg/m^3)	1.224	1.216	1.208	1.201
Absorption coefficient (mm^{-1})	3.881	3.854	3.829	3.807
F(000)	1488	1488	1488	1488
Crystal size (mm^3)	0.366 x 0.162 x 0.095	0.366 x 0.162 x 0.095	0.366 x 0.162 x 0.095	0.3664 x 0.1623 x 0.0946
Theta range for data collection	3.630 to 65.468°.	3.619 to 65.535°.	3.609 to 65.462°.	3.600 to 65.490°.
Index ranges	-19<=h<=19, -21<=k<=22, -14<=l<=14	-19<=h<=19, -21<=k<=22, -14<=l<=14	-19<=h<=19, -21<=k<=22, -14<=l<=14	-19<=h<=19, -21<=k<=22, -14<=l<=14
Reflections collected	33432	33749	33953	34219
Independent reflections	6484 [R(int) = 0.0316]	6542 [R(int) = 0.0335]	6581 [R(int) = 0.0336]	6600 [R(int) = 0.0357]
Completeness to θ = 65.5°	99.5 %	99.7 %	100.0 %	99.6 %
Absorption correction	Analytical	Analytical	Analytical	Analytical
Max. and min. transmission	0.852 and 0.639	0.697 and 0.382	0.852 and 0.4	0.852 and 0.639
Refinement method	Full-matrix least-squares on F2	Full-matrix least-squares on F2	Full-matrix least-squares on F2	Full-matrix least-squares on F2
Data / restraints / parameters	6484 / 12 / 436	6542 / 12 / 436	6581 / 12 / 436	6600 / 17 / 436
Goodness-of-fit on F2	1.064	1.042	1.057	1.049
Final R indices [I>2σ(I)]	R1 = 0.0384, wR2 = 0.0976	R1 = 0.0393, wR2 = 0.1019	R1 = 0.0399, wR2 = 0.1022	R1 = 0.0417, wR2 = 0.1088
R indices (all data)	R1 = 0.0507, wR2 = 0.1064	R1 = 0.0532, wR2 = 0.1119	R1 = 0.0555, wR2 = 0.1131	R1 = 0.0585, wR2 = 0.1211
Extinction coefficient	n/a	n/a	n/a	n/a
Largest diff. peak and hole (e.Å^{-3})	0.424 and -0.352	0.407 and -0.351	0.383 and -0.318	0.407 and -0.302
**Selected Bond Distances**				
Co1 - O2 (Å)	1.9306(13)	1.9663(14)	2.0017(14)	2.0344(16)
Co1 - N1 (Å)	1.9943(17)	2.0339(18)	2.0763(18)	2.112(2)
Co2 - O3 (Å)	1.8807(13)	1.8804(13)	1.8817(13)	1.8817(14)
Co2 - O4 (Å)	1.8810(13)	1.8816(13)	1.8829(14)	1.8830(15)
Co2 - N2 (Å)	1.9533(18)	1.9531(18)	1.9538(19)	1.954(2)
O4 - C20 (Å)	1.328(2)	1.327(2)	1.328(2)	1.325(2)
O3 - C21 (Å)	1.328(2)	1.328(2)	1.325(2)	1.326(2)
O2 - C6 (Å)	1.322(2)	1.313(2)	1.307(2)	1.297(2)
O1 - C1 (Å)	1.319(2)	1.312(2)	1.301(3)	1.294(3)

CSD survey

The Cambridge Structural Database (CSD) was analyzed using the ConQuest tool to investigate the correlation between pyridine plane torsion and Co–N bond distances. To find the queries of the family of Co(dioxolene)$_2$(PyL)$_2$ complexes, the following setup was searched:

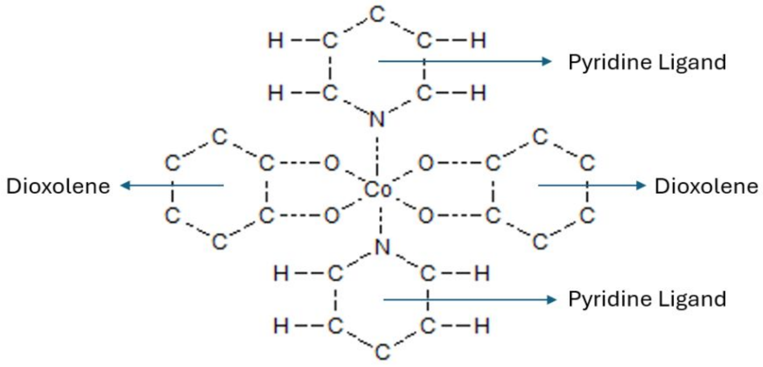

Figure S1: Search structure in in CSD database

Structures obtained from the ConQuest survey are shown Table S4. Complexes highlighted in yellow exhibit ligands that are not coplanar, making analysis of torsional effects inapplicable. These structures were therefore excluded from subsequent fitting procedures.

Table S4: CCD codes of the surveyed structures

ACIKEG	BIKZUT08	CAYSEE03	EJUNAA	GITVAK	LUTKER	PUTFAM03	WUQMOL
ACIKIK	BIKZUT09	CAYSEE04	EJUNAA01	ILEDOU	MUJJIM	PUTFAM03	XAJBAO
ACIKOQ	BIKZUT10	CAYYIO	EJUNAA02	KACCOI	MUJJIM01	PUTFAM04	XEVGAK
BIKZUT	BIKZUT11	DICKOT	EJUNAA03	KACCOI01	MUJJIM02	PUTFAM04	YEVMIW
BIKZUT01	BILBOQ	DICKUZ	EJUNEE	KEHJAL	MUJMEL	PUTGAN	YUWXIY
BIKZUT02	BILBOQ01	DICLEK	EJUNOO	LAXQIO	PUTFAM	PUTGER	YUWXIY01
BIKZUT03	BILBOQ02	DICLIO	EJUNUU	LAXQIO	PUTFAM	PUTGER	YUWXOE
BIKZUT04	CAYRUT	DICLIO	EJUPAC	LAXQUA	PUTFAM01	PUTGER01	YUWXUK
BIKZUT05	CAYSEE	DICLOU	ESUNUD	LOKPEI	PUTFAM01	PUTGER01	YUWYAR
BIKZUT06	CAYSEE01	EJUMON	ESUPAL	LUTKAN	PUTFAM02	RERHUU	YUWYEV
BIKZUT07	CAYSEE02	EJUMUT	GITTUC	LUTKAN	PUTFAM02	RERZIZ	YUWYIZ

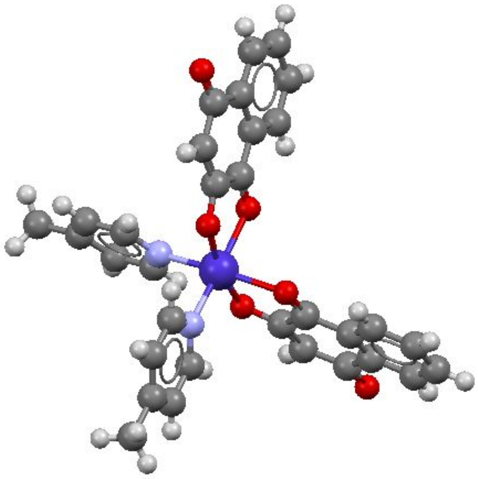

Figure S2: One of the structures removed from the analysis. CSD code: XAJBAO

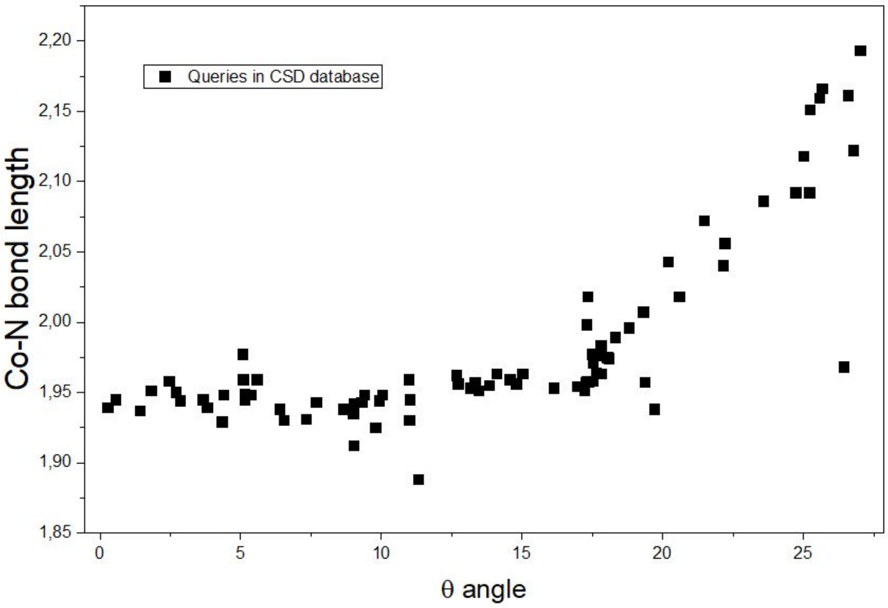

Figure S3: Plot of Co-N distance as function of the torsion angle for the 78 Co(dioxolene)$_2$(PyL)$_2$ surveyed structures.

To characterize the correlation between these parameters, an exponential fitting model was initially tested but subsequently discarded. The data were best described using two distinct linear regimes. To determine the optimal partition of data points between the two regimes, we employed an approach that minimized the mean squared error. Each partitioning scheme yielded different slopes for the linear fits.

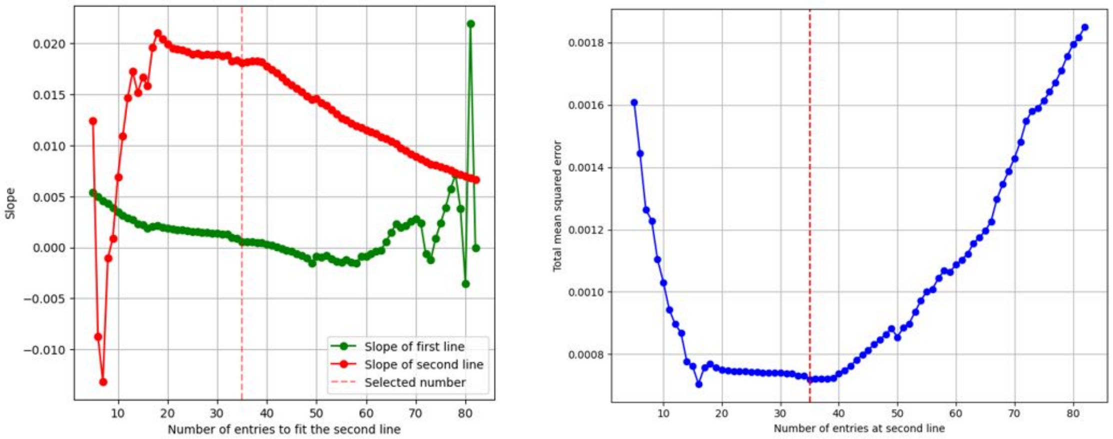

Figure S4: Slope fitting procedure os C-N diusatcnes if the 78 Co(dioxolene)$_2$(PyL)$_2$ surveyed structures.

Outlier discussion

The survey displayed in Figure 5 reveals the presence of an outlier. This complex (CSD code: YUWYIZ) deviates from the pattern because it exhibits Co–N bond distances characteristic of the ls-Co^{3+} state. However its torsion angle exceeds the critical value of 16.5°, which is atypical for this spin state. This discrepancy is related with short contacts in the crystal packing, which causes significant intermolecular interactions, as evidenced by red areas on the Hirshfeld surface. In this specific case, the torsion is not related to the spin state but with these intermolecular effects. The most relevant interaction appears to occur between a dioxolene oxygen atom and a hydrogen atom bonded to a ligand on a neighboring molecule.

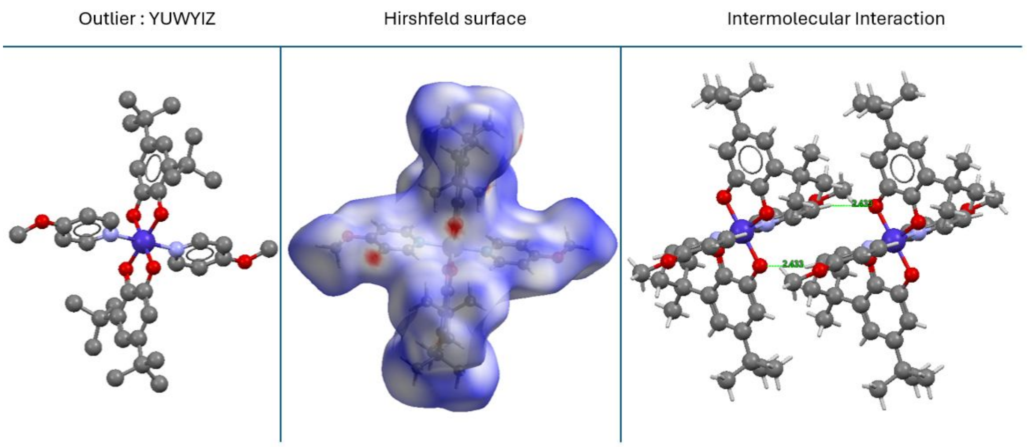

Figure S5: YUWYIZ structure indicating the intermolecular interactions.

C-N and C-O bond distance analysis for C-2:1P STRUCTURE

Co-L (L = N and O) distance as function of temperature upon both heating and cooling for different units of the **C-2:1P** structure characterized using SCXRD data. As evidenced, only **C-2:1P (A)** displays the VT interconversion thermally induced as indicated by the change in the Co-N bond distances.

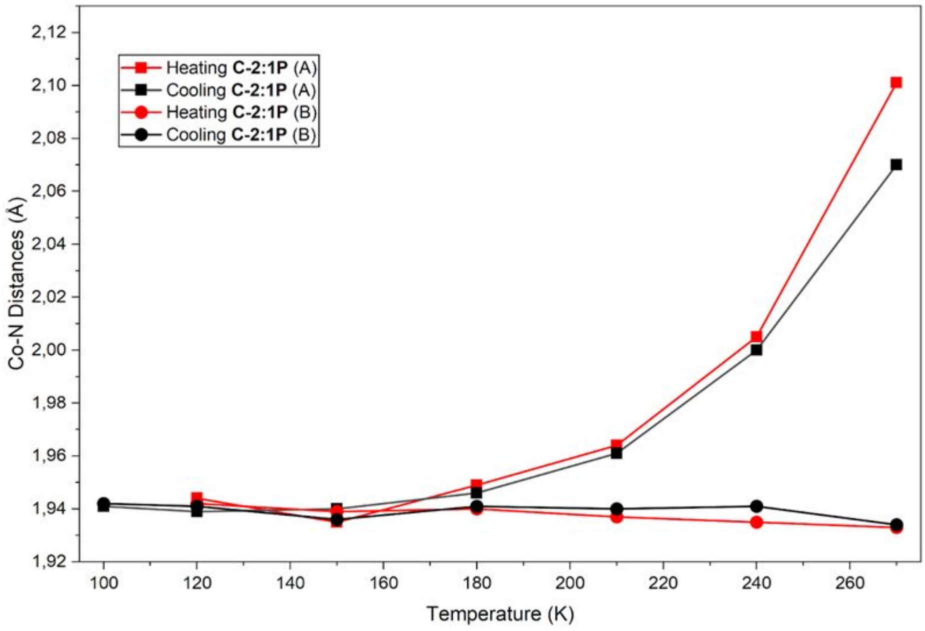

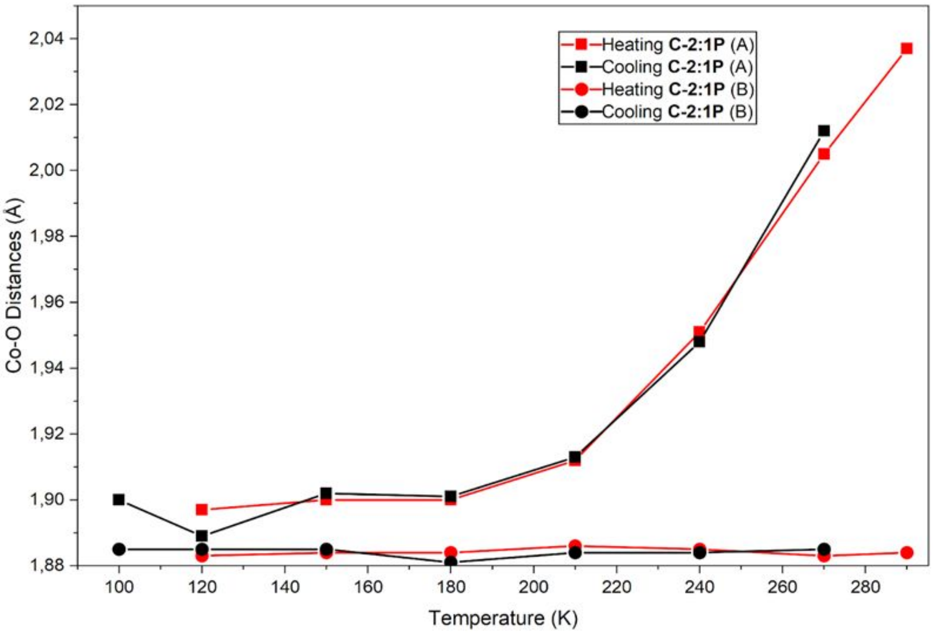

Figure S6: C-N and C-O bond distance for the **C-2:1P** structure

Torsion θ angle in Py plane as function of temperature upon both heating and cooling highlighting correlation between θ and VTI for tautomeric unit (A) of the **C-2:1P** structure.

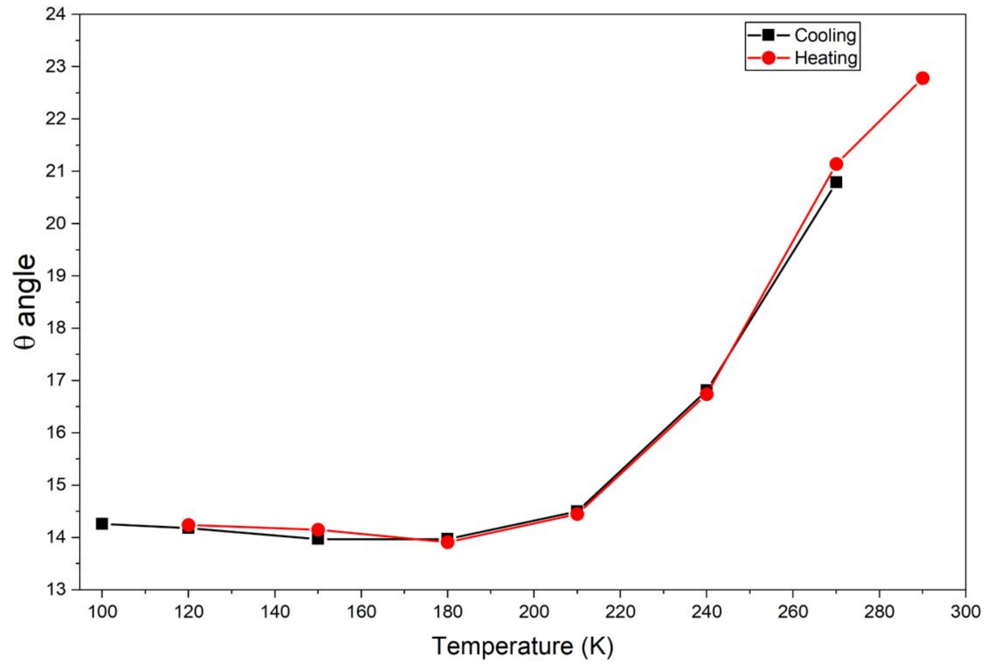

Figure S7: Torsion θ angle as function of temperature for the tautomeric unit (A) of the **C-2:1P** structure.

Across all compounds analysed in CSD database the torsion above critical angle is only displayed toward the nearest tert butyl groups considering the queries formed by 3,5-di-tert-butyl-o-dioxolenes. The hydrogens bonded to these tert-butyl groups seems to favor single bond preferential position due the interaction between them and the nearest oxygen, which is the same interacting with PyL.

DFT results

Table S5: Comparison between calculated and experimental bond lengths for cobalt in each spin state. The obtained values also suggest that despite its delocalization the single bond is preferentially in Co-O2 than Co-O1 in *hs*-Co^{2+}(Figure 9), causing the slightly larger bond distances.

	ls-Co^{3+}		***hs-Co^{2+}***	
	Calc	*Exp*	*Calc*	*Exp*
Co-O1 (Å)	1.86	1.88	1.98	2.00
Co-O2 (Å)	1.87	1.88	2.04	2.03
Co-N (Å)	1.89	1.95	2.06	2.11

hs-Co^{2+}

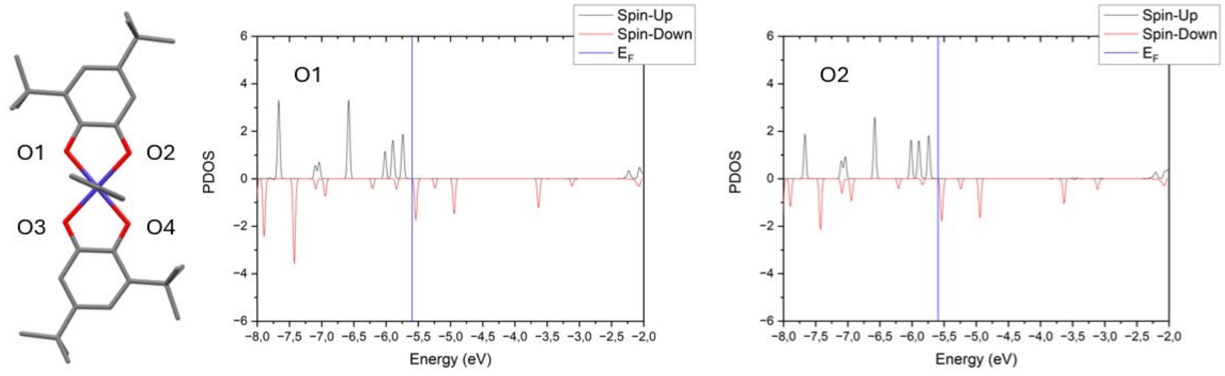

ls-Co^{3+}

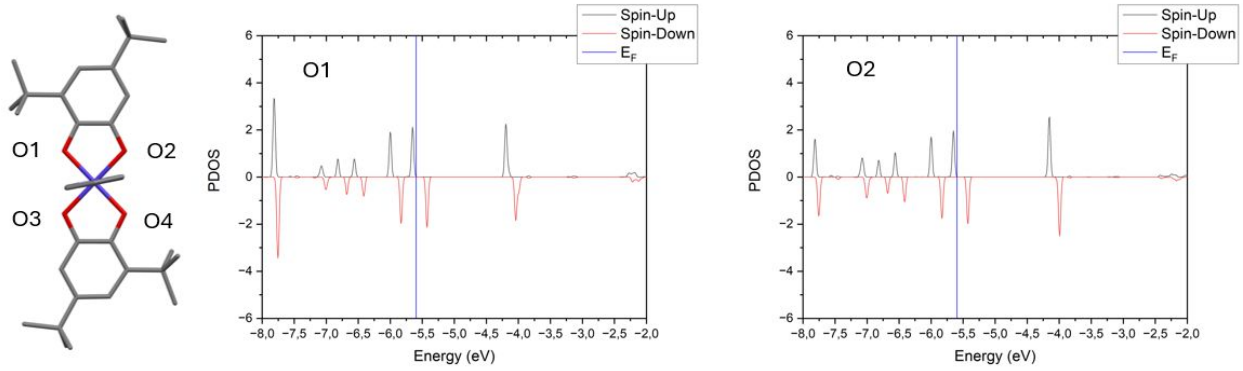

Figure S8: Projecting the density of states over equivalent oxygens atoms we can see how it changes as a function of the energy around Fermi level for *ls*-Co^{3+} in comparison with *hs*-Co^{2+}.

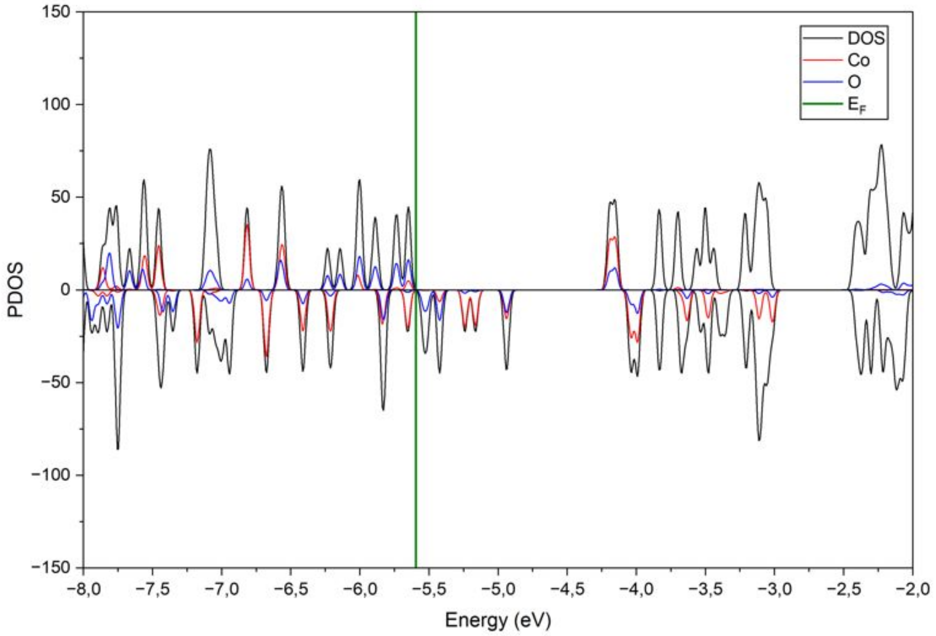

Figure S9: A comparison among the Density of States around the Fermi level with it projected over Co and O atoms, allowing us to see which bands are related with each atom.

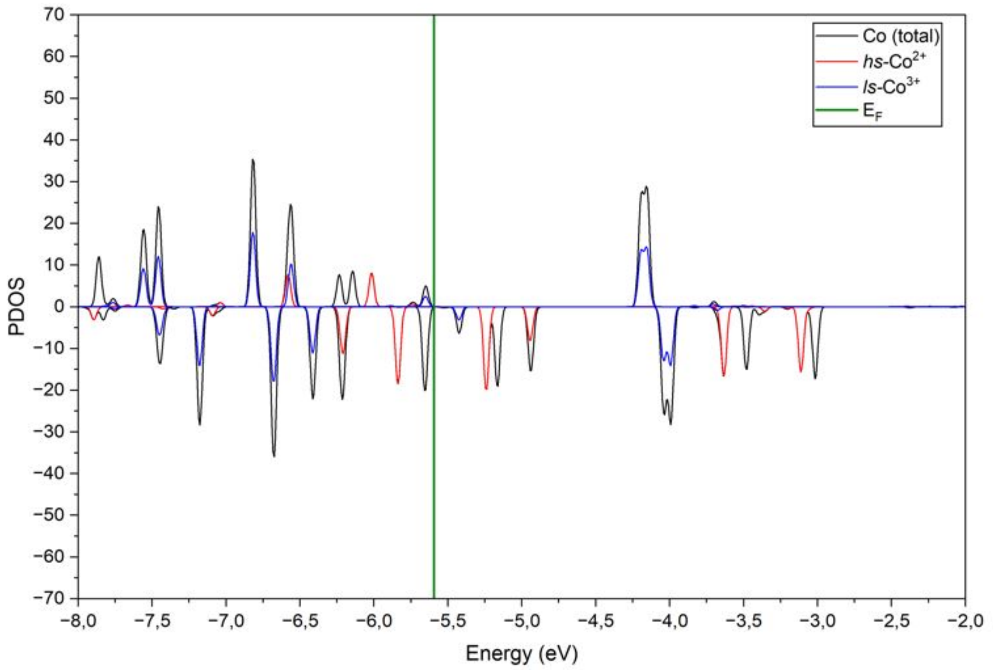

Figure S10: A comparison among Co Projected Density of States with Co atoms in different spin-states.

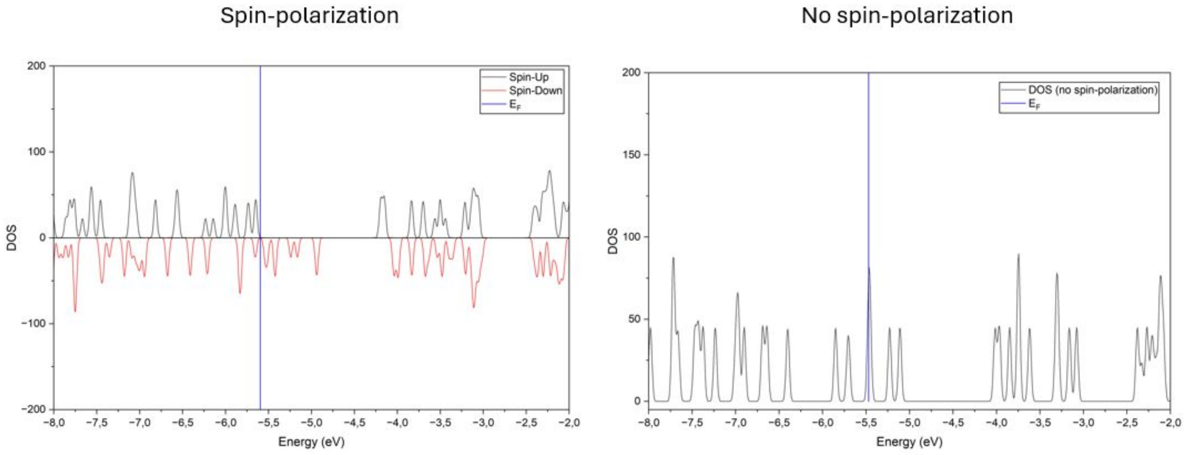

Figure S11: The Stoner model establishes that the occurrence of ferromagnetic ordering is directly related to the spontaneous splitting of spin-up and spin-down bands due to exchange interactions. The Stoner criterion determines that if spin polarization is not included in the calculation, a ferromagnetic arrangement is expected when the product of the density of states at the Fermi level and the Hubbard parameter is higher than 1. Although this analysis was not carefully conducted and is not in the scope of the present work, the significant density of states observed for this compound suggests potential ferromagnetic character.
